# Nano-imprinting of refractive-index-matched indium tin oxide sol–gel in light-emitting diodes for eliminating total internal reflection†

**DOI:** 10.1039/c8ra06773b

**Published:** 2018-11-01

**Authors:** Sungjoo Kim, Chul Jong Yoo, Jae Yong Park, Sangwon Baek, Won Seok Cho, Jong-Lam Lee

**Affiliations:** Department of Materials Science and Engineering, Pohang University of Science and Technology (POSTECH) Pohang 790-784 Korea jllee@postech.ac.kr; Division of Advanced Materials Science, Pohang University of Science and Technology (POSTECH) Pohang 790-784 Korea

## Abstract

Refractive-index (RI)-matched nanostructures are implemented in GaN-based light-emitting diodes (LEDs) for enhancing light output efficiency. The RI-matched indium tin oxide (ITO) nanostructures are successfully implemented in GaN-based lateral LEDs by using ITO sol–gel and nanoimprint lithography. The ITO sol–gel nanostructures annealed at 300 °C have RI of 1.95, showing high transparency of 90% and high diffused transmittance of 34%. Consequently, the light output power in LEDs with the RI-matched nanostructures increases by 8% in comparison with that in LEDs containing flat ITO. Ray tracing and finite-difference time-domain (FDTD) simulations show that the RI-matched nanostructures on the transparent current spreading layer dramatically reduce Fresnel reflection loss at the interface of the current spreading layer with the nanostructure and extract confined waveguide lights in LEDs.

## Abstract 2

Refractive-index (RI)-matched nanostructures are implemented in GaN-based light-emitting diodes (LEDs) for enhancing light output efficiency.

## Graphical abstract

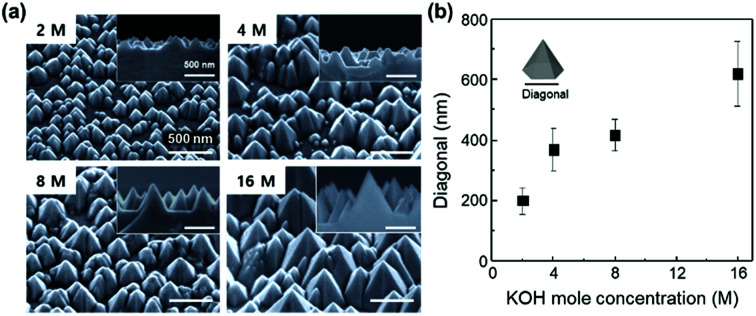

## Introduction

Group III-nitride light-emitting diodes (LEDs) are promising candidates for next-generation lighting sources because of their high efficiency, long life, and environmental friendliness.^1–3^ To replace conventional light sources with LEDs, the quantum efficiency of LEDs should be further increased. Quantum efficiency is determined using internal quantum efficiency (IQE) and light extraction efficiency (LEE). An IQE value of nearly 80% has been reported,^4,5^ but there is still much room for enhancement of LEE. This is due to the fact that most of the generated photons from the active layer are captured by LEDs because of total internal reflection (TIR) at the interface of semiconductor with air.^6,7^ To solve this problem, a number of solutions based on geometrical optics have been proposed including integration of two-dimensional (2D) photonic crystal (PC) patterns,^8–12^ growth of nano-wires such as zinc oxide (ZnO),^13–19^ and nano-patterns using electron beam lithography.^20,21^ However, these methods are intrinsically expensive, exhibit low-throughput and are limited to small-area processing. Also, surface roughening by dry etching can produce defects in the GaN layer, leading to reduction of the overall efficiency of LEDs.^22–24^

Nano-imprint lithography offers significant improvements in the high throughput fabrication of surface patterns over large areas^25–27^ while preserving the integrity of LEDs. However, reported imprinted materials, such as SU-8 and polydimethylsiloxane (PDMS), are polymers with lower RI than that of the top transparent current spreading layer indium–tin–oxide (ITO). Therefore, TIR and Fresnel reflection can occur at the interface of ITO with soft resin, resulting in the reduction in the efficiency of LEDs.^28,29^ These problems can be solved by employing an RI-matched layer, namely, ITO nanostructure on ITO-coated LEDs.

In this study, we report a way to implement novel hexagonal pyramid-shaped nanostructures using an RI-matched ITO sol–gel on the transparent current spreading ITO layer. Through the RI-matched material with nanostructures, the following results are successfully achieved: (1) surface texturing of the top layer and (2) reduced Fresnel reflection loss at the interface of the nanostructure with ITO. Implementing the RI-matched nanostructures onto LEDs improves light output power in comparison with those with flat ITO. The dramatic performance enhancement is due to effective elimination of TIR, which is consistent with theoretical calculations using ray tracing and finite-difference time-domain (FDTD) simulations.

## Experimental section

### Preparation of ITO sol–gel solution

An ethanol-based ITO sol was prepared using tin(ii) chloride (SnCl_2_; Sigma-Aldrich) and indium(iii) nitrate hydrate (ln(NO_3_)_3_·H_2_O; Sigma-Aldrich) as a precursor. Initially, tin(ii) chloride and indium(iii) nitrate hydrate were added to absolute ethanol and mixed. The solution was stirred for two hours at room temperature. Subsequently, small amounts of acetylacetone (C_5_H_8_O_2_; Sigma-Aldrich) were added to the above solution with stirring. The Sn contents were calculated to give the atomic percentage of Sn (*i.e.*, atoms Sn/(atoms Sn + atoms In)) of 10%. It was found that the resistivity of ITO films was minimum at 10 at% (Sn relative to In). The reaction was kept under mechanical stirring (600 rpm) for 4 h at 78 °C. The viscosity was controlled by volatilizing the ethanol solvent.

### Fabrication of nano-imprint mold

To fabricate a replica mold, the surface of master mold was functionalized by a liquid-phase coating of trichloro(1*H*,1*H*,2*H*,2*H* perfluorooctyl)-silane (97%, Sigma-Aldrich) for 20 min to form a self-assembled monolayer (SAM), which has a functional unit of –CF_3_ as a releasing layer. The stamp was rinsed with ethanol and acetone in sequence after the fluorosilane SAM treatment. The fluorinated releasing layer lowers the surface energy of stamp and assists in separation. After the anti-adhesion process, the master mold was pressed onto the UV-curable polymer (Ormoclear, MicroResist Tech., Germany) that was dropped on the substrate (polycarbonate (PC) film). Then, the polymer was cured for 15 min under UV light (90 mW cm^−2^) under pressure (20 bar). To improve adhesion, the substrate was treated with UV-O for 20 min under a power density of 30 mW cm^−2^. The master pattern was successfully transferred to the UV-curable polymer replica mold on the substrate and then, this replica mold was used in imprinting ITO sol–gel.

### Thermal nano-imprint lithography (NIL) of ITO sol–gel

The ITO sol solution was spin-coated onto a glass substrate at 1000 rpm for 30 s. The replica mold was placed onto the spin-coated substrate and an imprinting pressure of 100 bar was applied at 150 °C for 30 min. During the imprinting step, the ITO sol filled the cavity of replica mold completely, and the organic solvent of ITO sol was absorbed onto replica mold and removed. Consequently, the ITO sol was converted to the ITO gel during the imprinting step, resulting in the production of nanopatterns of ITO gel.

### Device fabrication

The ITO sol–gel was coated on a GaN LED wafer, on which a nanopattern was produced using nanoimprint lithography. An active region was defined by inductively coupled plasma using Cl_2_/BCl_3_ gas. For ohmic contact formation on p-type GaN, Ni (50 Å) and Au (100 nm) metals were deposited in sequence. The Cr/Au n-type ohmic contact was deposited on n-type GaN. All metals were deposited on p-type and n-type GaN by electron beam evaporation under the pressure of 2 × 10^−7^ torr, followed by removing the metals deposited on photoresist.

### Measurements

High-magnification scanning electron microscopy (SEM) images were obtained using a PHILIPS XL30S instrument with an accelerating voltage of 10 kV. The RI and layer thickness were measured using variable angle spectroscopic ellipsometry. The X-ray photoelectron spectroscopy (XPS) was performed using 8A beam line, Pohang Accelerator Laboratory (PAL) with a base pressure of 5 × 10^−10^ torr. The crystallinity of ITO films was identified using a Rigaku D/Max-2500 X-ray diffractometer. The UV-visible spectra were recorded on a Perkin Elmer Lambda 750S UV/VIS spectrophotometer with a wavelength range from 400 nm to 800 nm. Light output–current–voltage (*L*–*I*–*V*) characteristics of LED were measured using an Agilent B2902A precision source-measurement unit.

## Results and discussion

To produce nanostructured ITO sol–gel, various sizes of GaN master molds were fabricated by using photochemical etching with KOH solution. The photochemical etching (PCE) process included two simultaneous chemical reactions of oxide formation and oxide dissolution. The hydroxide ions (OH^−^) in KOH solution were adsorbed on the GaN surface and subsequently reacted with Ga atoms, as described by following the reaction:^30^
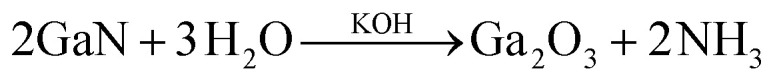


**Fig. 1:**
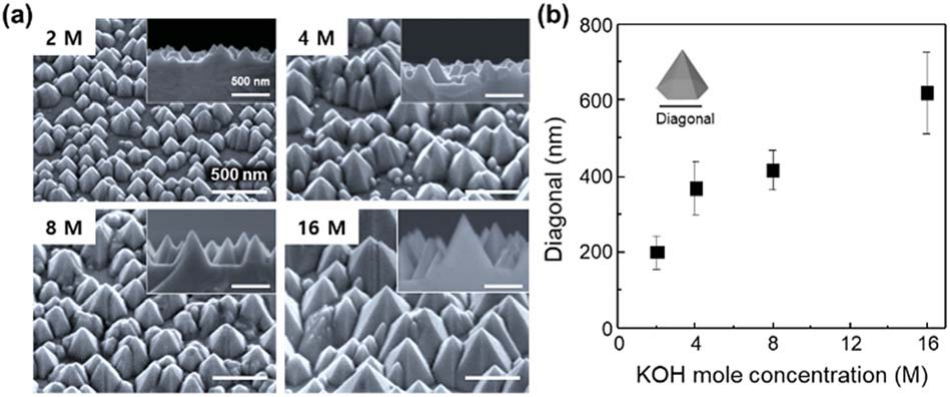
(a) SEM images of photochemically etched GaN with 2, 4, 8, and 16 M-KOH for master mold. (b) Average value of bottom diagonal of hexagonal pyramids expressed as a function of KOH molar concentration.

KOH acts as a catalyst and solvent for resulting Ga_2_O_3_. As the stages of (b) to (e) are repeated, N-polar GaN can be etched (Fig. S1†). Therefore, PCE produces hexagonal pyramid shapes with a fixed side-wall angle of 31.6° because {10-1-1} surface of GaN has the lowest surface energy and smallest number of bonds.^31^ The size of a nanostructure can be easily controlled by molar concentration of KOH (Fig. 1a). As the molar concentration increases from 2 to 16 M, the average bottom diagonal increases from 180 to 590 nm (Fig. 1b). The nanostructure with longer diagonal is expected to effectively scatter incident light based on the grating equations.^32^ Thus, the size of the optimal diagonal is set to 600 nm.

**Fig. 2:**
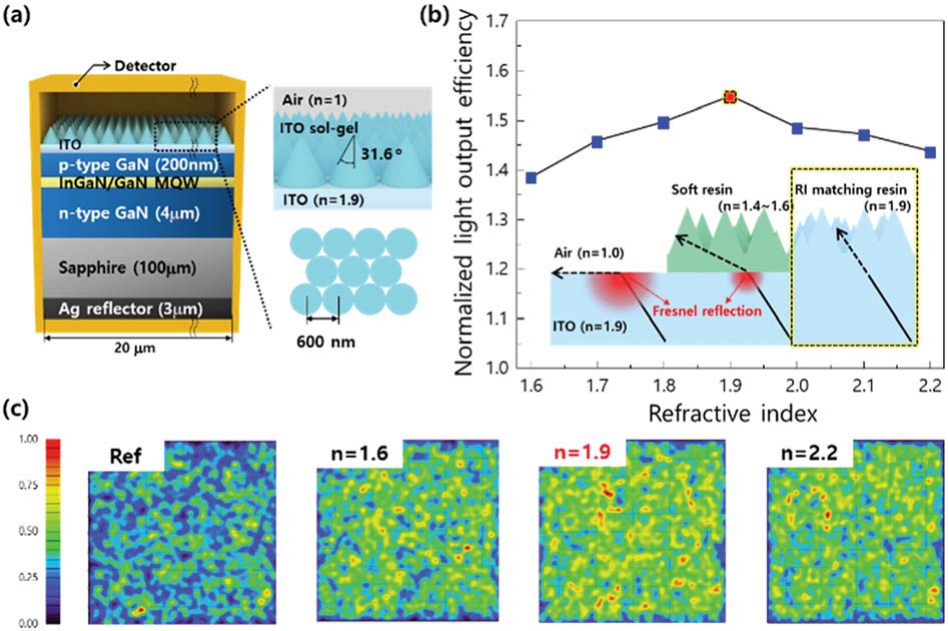
(a) Schematic illustration of the nano-imprinted LEDs with pyramid-shaped ITO nanostructures formed by RI-matched resin. (b) Calculated light extraction efficiency with refractive index; the inset shows schematic explanation of the mechanism for improving light extraction with different RI layers. (c) Calculated far-field intensity with nanostructures having different refractive indices by ray-tracing (ref, bare ITO). The wavelength was fixed at 450 nm.

The theoretical Monte Carlo ray tracing simulation was conducted to examine the effect of RI on the light output efficiency of LEDs. Fig. 2a shows schematic illustration of a lateral LED structure designed for the calculation of light output. It is assumed that lights are generated randomly within the active region and emitted in all directions.^33^ The nanostructure on the planar ITO layer has a diagonal of 600 nm and a side wall angle of 31.6° based on the shape of photochemically etched GaN (Fig. 1). The light output efficiency is calculated as a function of the RI of nanostructures and then normalized to that of planar ITO layer (Fig. 2b). The normalized light output efficiency increases as RI increases and it maximizes at RI = 1.9. Further increase in RI decreases the light output efficiency. Far-field intensity at the top of LEDs is calculated with refractive indices by ray-tracing. The light output is maximized at RI = 1.9 (Fig. 2c). It is remarkable that the light output intensity is maximized when the RI of the nanostructured layer is similar to that of transparent current spreading ITO layers (RI = 1.95 at *λ* = 450 nm). This observation can be explained by the maximized efficiency of LEDs originating from the elimination of Fresnel reflection loss at the nanostructured layer/ITO interface.

**Fig. 3:**
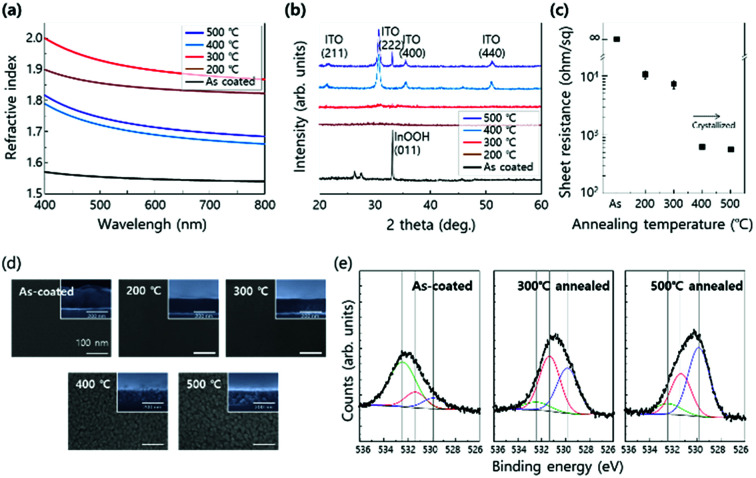
(a) Refractive index, (b) GIXRD and (c) sheet resistance results of ITO sol–gel films as functions of the annealing temperature. (d) SEM images of ITO sol–gel films. At 400 °C and 500 °C, ITO sol–gel films show porous morphology. The inset shows the cross-sectional images. (e) X-ray photoelectron spectra (O(1s) peak) of as-coated, 300 °C, and 500 °C annealed ITO sol–gel films. (∼530 eV: M–O–M lattice oxygen, ∼531 eV: M–OH metal hydroxide oxygen, and ∼532 eV: adsorbed oxygen species) The annealing condition was fixed in an N_2_ ambient for 3 min.

Fig. 3a shows the change in RI of ITO sol–gel films as a function of annealing temperature. The annealing condition was fixed in an N_2_ ambient for 3 min. At an as-coated condition, the RI values were measured to be lower than 1.6, resulting from low density of the film due to polymeric property of the sol phase.^34–36^ As the annealing temperature increased to 200 °C, the RI values increased, but they were still lower than 1.95 (RI_sputtered ITO_ ≈ 1.95 at *λ* = 450 nm). RI increased to the value of sputtered ITO as the annealing temperature increased to 300 °C. Further increase in the annealing temperature up to 500 °C drastically decreased the RI value to below 1.8. To explain the change in RI with annealing temperature, the crystallinity of ITO sol–gel was examined as a function of annealing temperature. Grazing incidence angle X-ray diffraction (GIXRD) analysis revealed that the film maintained amorphous state up to 300 °C, as shown in Fig. 3b. When the annealing temperature increased to 400 °C, the film began to crystallize, and the sheet resistance abruptly decreased (Fig. 3c). Scanning electron microscopy (SEM) images clearly showed the change in morphology of thermally transformed ITO sol–gel, as shown in Fig. 3d. At the as-coated state, the surface morphology was maintained to be flat up to 300 °C, but it changed to rough. The cross-sectional view of the film revealed that the porosity of ITO sol–gel layers increased at temperatures higher than 400 °C, as shown in the inset of Fig. 3d. This change lowered the effective RI (Fig. 3a and d).^6^ X-ray photoelectron spectroscopy (XPS) showed that the O1s spectrum of ITO sol–gel could be deconvoluted into three kinds of peaks centered at 532.3, 531.1, and 530.1 eV, which were ascribed to adsorbed oxygen, bulk In–OH, and lattice oxygen of metal–oxygen–metal (M–O–M), respectively. The as-coated films showed a significant amount of adsorbed oxygen species from the solvent because of incomplete transformation from gel to sol. At the annealing temperature of 300 °C, the In–OH peak has the largest ratio among the three peaks, indicating incomplete formation of the oxide lattice. However, at the annealing temperature of 500 °C, the peak of M–O–M lattice oxide is dominant, which was due to crystallization of ITO with efficient condensation and subsequent densification. This result is in agreement with SEM (Fig. 3d and S2†) and GIXRD (Fig. 3e) results. Based on the enhancement in light output efficiency of LED in Fig. 2, the optimal annealing temperature was determined to be 300 °C due to smooth morphology and RI of 1.95, which were consistent with the observations for the transparent current spreading layer of ITO. Further information on chemical elements of the annealed ITO sol–gel is described in Fig. S3.†

**Fig. 4:**
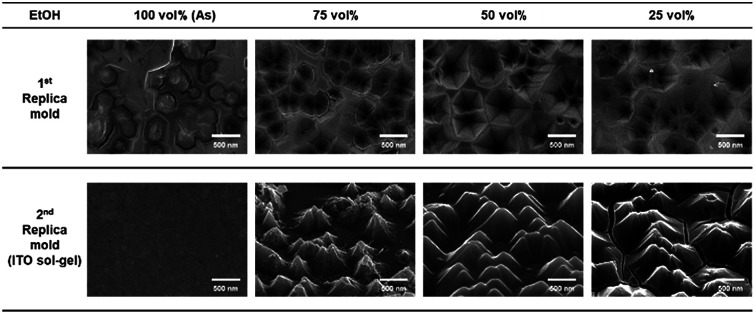
SEM images of 1^st^ replica mold and 2^nd^ replica mold as a function of remaining ethanol solvent after volatilization.

The fabrication flow of nanoimprint lithography of ITO sol–gel is described in detail in Fig. S4.† First, the nanostructures of the photochemically etched GaN master mold were transferred to a polymeric 1^st^ replica mold, Ormoclear, under ultra-violet light and pressure of 1 kgf cm^−2^. Then, the nanostructures of the 1^st^ replica mold were transferred to ITO sol–gel with thermal nanoimprint lithography. During the imprinting of ITO sol–gel, annealing temperature was set to 150 °C to protect thermal-induced damages in the polymeric 1^st^ replica mold. Then, the post annealing process at 300 °C was used to match RI of ITO sol–gel to that of the transparent current spreading layer (Fig. 3a). By controlling the viscosity of sol–gel solution, the pyramid shape could be transferred from the 1^st^ replica mold. The viscosity of the sol–gel solution was controlled by volatilization of ethanol (Fig. 4). As the ethanol solvent volatilized, the viscosity of the ITO sol–gel solution increased because ethanol with low viscosity (0.001 Pa s) was vaporized. At the initial state without volatilization, the nanostructures on 1^st^ replica mold were not completely transferred to ITO, leaving a planar structure on the ITO. This is because the highly viscous sol–gel solution led to insufficient peel-off between the 1^st^ replica mold and the nanostructured ITO. As the amount of volatilized ethanol increased, the surface of ITO began to exhibit a nanostructure after nanoimprinting. However, when the concentration of remaining ethanol was 25 vol%, cracks were observed on the ITO nanostructures. Thus, the ethanol concentration of 50 vol% was chosen as an optimal concentration for volatilization.

**Fig. 5:**
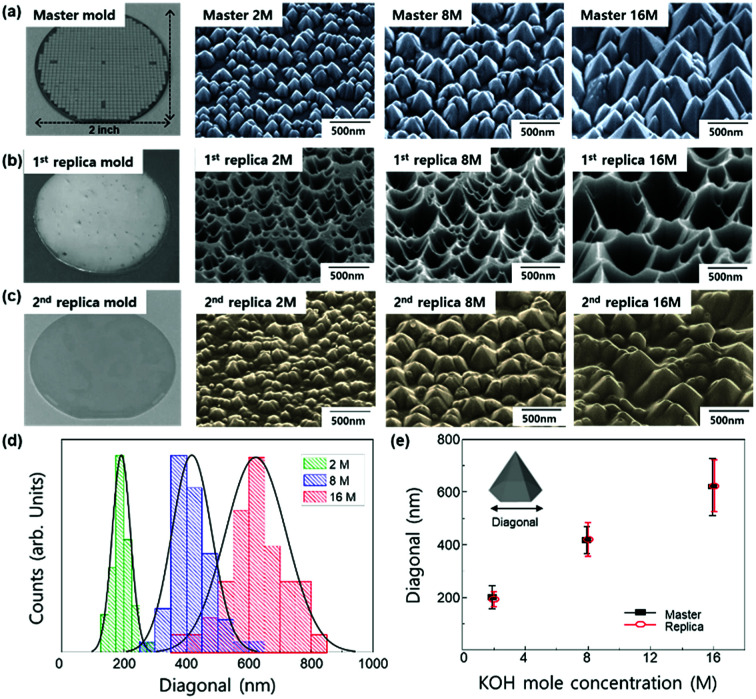
Photographs and SEM images of nano-structures shaped as different hexagonal pyramid patterns. (a) Master mold of photochemically etched GaN with 2 M, 8 M, and 16 M-KOH. (b) Inverted-patterned 1^st^ replica polymer mold imprinted from the master mold. (c) 2^nd^ replica mold of ITO sol–gel imprinted from the 1^st^ replica mold. (d) Distribution of the length of diagonal in the 2^nd^ replica ITO sol–gel. (e) Average diagonal of the nanostructure on the 2^nd^ replica ITO sol–gel.

To investigate the effect of nanostructure on the enhancement of LEE in LEDs, a nanostructure was produced on the ITO sol–gel coating on LED using the nano-imprint lithography. The pyramid-shape master mold with different feature sizes was produced by PCE using different concentrations of KOH solution (Fig. 5a). After PCE with KOH solution, hexagonal pyramids were formed on the surface of GaN by preferential etching of the GaN {10-1-1} surface because of its lowest surface energy and smallest number of bonds.^31^ Because the chemical etching rate depends on the molar concentration of KOH, it made the bottom diagonal increase from 180 to 590 nm with the increment of mole concentration from 2 to 16 M (Fig. 1b). The imprinting technique with a UV-curable precursor was employed to fabricate inverted-patterned 1^st^ replica polymer molds using a rigid master mold (Fig. 5b). The replica mold of 2 inch scale size was successfully demonstrated to implement the nanostructure pattern of the ITO sol–gel (Fig. 5c). The average bottom diagonals of replicas changed from 180 to 590 nm (Fig. 5d), corresponding to that of the master mold (Fig. 5e).

**Fig. 6:**
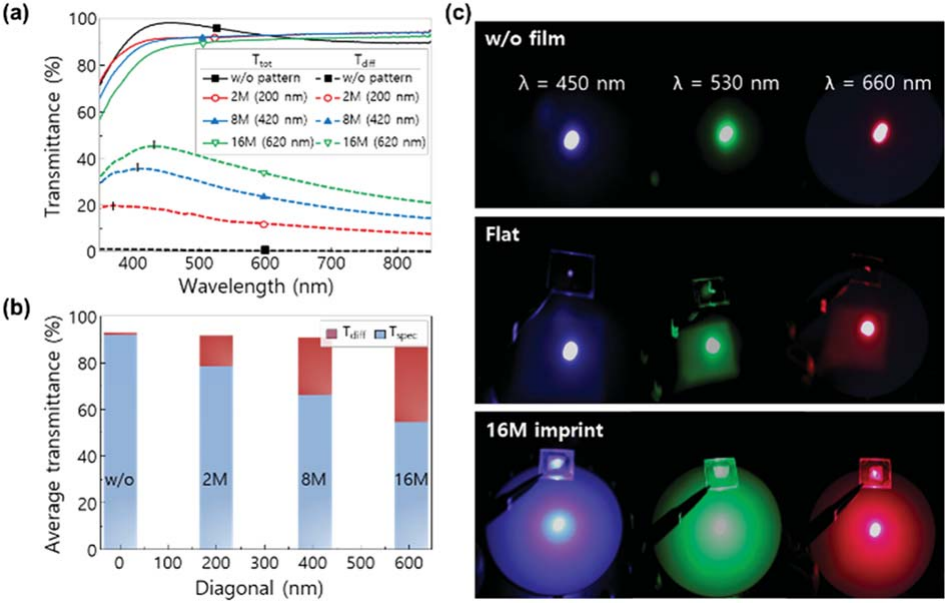
(a) Total (line) and diffused (dot) transmittance spectra of nano-imprinted ITO replica with different sizes of hexagonal pyramids as a function of wavelength. (b) Average diffusive and specular transmittance (380–860 nm) of nano-imprinted ITO replica as a function of average bottom diagonal. (c) Photographs of scattered light by nano-imprinted ITO replica using monochromatic laser with wavelengths of 460 nm (blue), 530 nm (green) and 660 nm (red). Light intensity was set constant at 60 mW cm^−2^.

To examine the optical scattering properties of the nano-imprinted structure, total transmittance and diffused transmittance were measured by a UV-visible spectrometer, as shown in Fig. 6a. The average total transmittance of flat film was measured to be 94.2%. As the nano-patterns were employed on top of ITO, the transmittance decreased to 92.5% for the 2 M imprinted sample, 92.3% for the 8 M imprinted sample, and 89.8% for the 16 M imprinted sample. The decrease in total transmittance could be explained by the grating equation.^32^ The nano-patterns allowed higher reflective diffraction order than transmissive diffraction order at a given wavelength range, leading to decrease in total transmittance. In flat ITO, the diffused transmittance was 0.74%, whereas it dramatically increased to 12.9% (2 M), 24.8% (8 M), and 34.3% (16 M) in the nano-imprinted samples. As shown in Fig. 6b, the average diffusive and specular transmittance (*λ* = 380–860 nm) of nano-imprinted ITO replica are calculated as a function of average bottom diagonal. As the diagonal of nano-structure increased, the average specular transmittance decreased, but the average diffusive transmittance increased. Scattering effect was not observed for the flat film, but the ring-pattern indicating the Rayleigh scattering effect appeared in 16 M nano-imprinted ITO (Fig. 6c). In addition, the dependence of wavelength on haze is remarkable. As the laser wavelength decreased from 660 nm (red) to 460 nm (blue), the scattered ring-pattern became larger and clearer. This observation provides evidence that the scattering behavior in the nano-imprinted samples is highly dependent on the size of nanostructures.

**Fig. 7:**
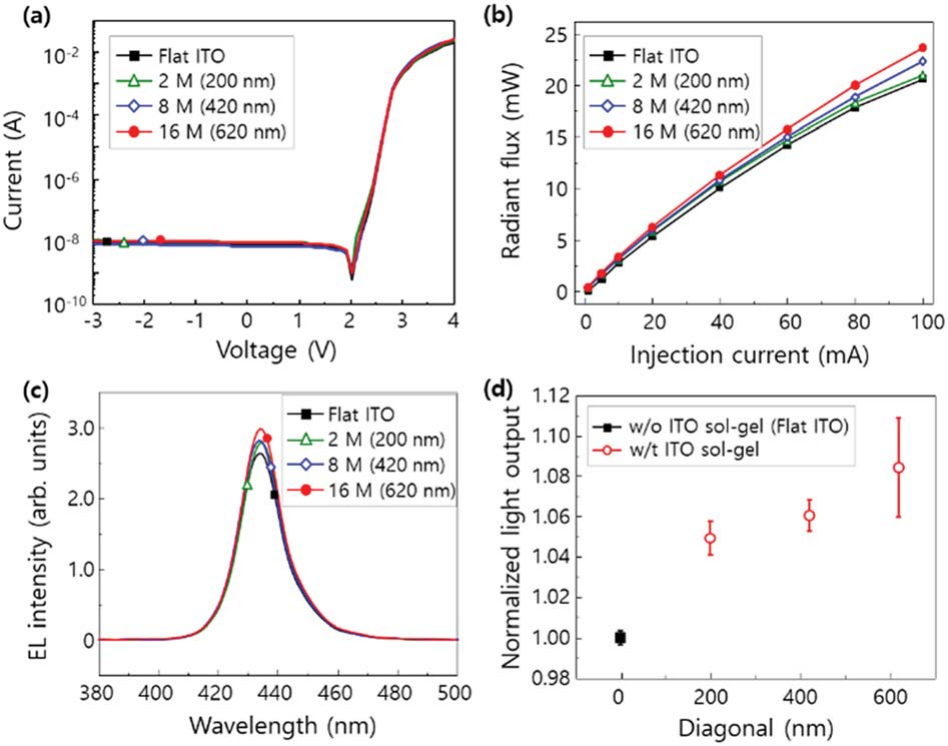
(a) Current–voltage (*I*–*V*) characteristics of LEDs. (b) Radiant flux of LEDs as a function of injection current (5–100 mA). (c) Electroluminescence (EL) spectra of LEDs with different surface structures at a 20 mA injection current: flat, pyramid-shaped nanostructures with different sizes formed using PCE. (d) Measured light output power of LEDs as a function of hexagonal pyramid size of a nanostructure.

The current–voltage (*I*–*V*) characteristics of LEDs are almost the same and independent of the size of nanopatterns for all samples (Fig. 7a). The reverse leakage currents at −3 V and the forward voltage at 20 mA mm^−2^ were measured to be in the range from 90 to 114 nA mm^−2^ and 3.74 to 3.92 V. The radiant flux of LEDs was also measured as a function of injection current (Fig. 7b). The largest nanostructure (16 M, diagonal = 620 nm) showed the maximized radiant flux for overall injection current. The electroluminescence (EL) spectra of LEDs with flat ITO surface and the nano-imprinted surface with various hexagonal pyramid structures were measured at an injection current of 20 mA (Fig. 7c). The EL intensity in the nano-imprinted ITO sol–gel LED increased with the increase in the diagonal of nanostructure. The light output efficiency as a function of diagonal of the nanostructures is plotted in Fig. 6d. In the ITO sol–gel imprinted LEDs, light output powers increased by 5% (2 M), 6% (8 M), and 8% (16 M) in comparison with that of flat ITO LED as the diagonal of the nanostructure increased. It is implied that ITO sol–gel imprinted nanostructures are useful for the enhancement in light output power of LEDs.

**Fig. 8:**
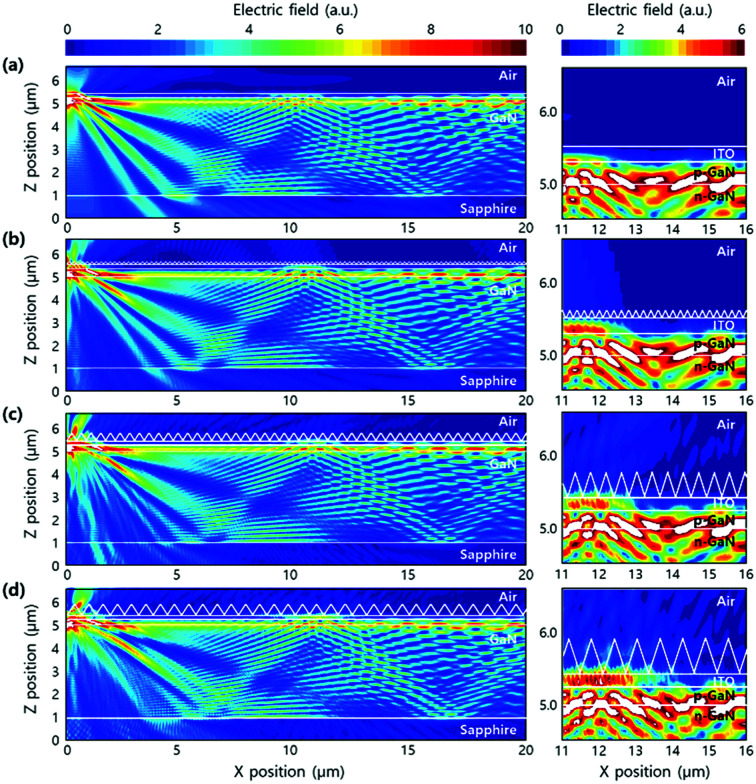
(Left) Calculated cross-sectional electric field distribution of the LEDs with (a) flat ITO structure, nanostructured ITO with (b) 2 M, (c) 8 M, and (d) 16 M KOH. (Right) Enlarged view of the electric field distribution between ITO and air.

To theoretically study the optical effect of ITO sol–gel on the light output efficiency of LEDs, two-dimensional finite-difference time-domain (2D FDTD) simulations were conducted. The boundary condition is given in the simulation. The periphery of LED was set to be a perfectly matched layer, at which all photons were absorbed, to avoid unwanted reflection at the layer surface. In the calculation, the dipole source with vertical and horizontal polarizations of 450 nm in the InGaN multi quantum well (MQW) was used. The calculations proceeded until the change of electric field was <0.01% per FDTD time steps to reach steady state. The discrete Fourier transform (DFT) monitor was used to obtain cross-sectional electric field distributions at the steady state. Planar and nanostructured ITO devices were designed for the calculation of electric field distributions. In the planar device, the electromagnetic wave was confined to the inside of LED due to the internal reflection at the ITO/air interface (Fig. 8a). However, in the nanostructured ITO LED, the electric field was observed at the outside of ITO, indicating the extraction of confined electric field (Fig. 8b–d). The intensity of electric field in the air increased with the size of nanostructure. As a result, the calculated light output efficiencies increased to 7.8% (2 M), 42.4% (8 M), and 68.0% (16 M) in comparison with that of the planar device. This is in accordance with the experimental results depicted in Fig. 7. Such improvements are because large patterns effectively scatter incident light, according to the grating equations.^28^

Based on a previous study on the improvement in light extraction efficiency of planar GaN-based LEDs,^37^ polystyrene nanospheres on ITO transparent electrodes were used as an etching mask to form the shape of an ITO nanodisk by inductively coupled plasma etching. A theoretical study was conducted on the shape of ITO nanodisk (diameter and height) to improve the light extraction efficiency. The geometry of ITO nanodisk was designed to satisfy the zero reflection condition by the transfer matrix method, leading to increased extraction of light from LED. However, there were some limitations in forming the ITO nanodisk. The nanosphere lithography for patterning ITO nanodisk is known to be limited in uniformly covering a large area. In the electrical properties of LEDs, no difference in forward voltage at 20 mA was observed with plasma etching, but there seemed to be some degradation at high forward voltages (>100 mA). This could be due to production of defects in p-GaN during plasma etching.^38,39^ In the meanwhile, nanoimprint lithography was employed, which is a novel way to form a nanostructure using ITO sol–gel on GaN-based LEDs. The advantages of this process include inhibition of etching-induced damages as well as easy formation of various nanostructures. The current–voltage characteristics in nanostructured LEDs are almost the same as those of the flat ones, but light output power increases by 12%. Therefore, it is concluded that ITO sol–gel-based methods increase the light extraction efficiency without any degradation in the electrical properties. The FDTD simulation provides evidence that the RI-matched nanostructures can maximize the efficiency of LEDs by eliminating the Fresnel reflection loss at the nanostructure/ITO interface. In addition, nanostructures with large diagonals are effective in enhancing light output efficiency based on the grating equations in FDTD simulation. We conclude that the nanostructure ITO sol–gel plays a critical role in increasing light output efficiency by inducing light scattering.

## Conclusions

In conclusion, we report a novel method of improving light output efficiency by implementing RI-matched ITO nanostructures on LEDs, thus opening the possibility of further improvement in optical devices. The RI-matched ITO sol–gel nanostructures can effectively eliminate the Fresnel reflection loss between transparent current spreading ITO layer and RI-matched ITO nanostructures. The sol–gel process enables the production of nanostructures on the ITO with large area of 2 inch ITO-coated GaN. The imprinted ITO sol–gel nanostructure increases the diffused transmittance from 0.74% to 34.3%. Consequently, ITO sol–gel-imprinted LEDs provide 8% enhanced light output power compared to the flat device. FDTD simulations show that the nanostructured ITO sol–gel extracts confine waveguided mode in the ITO film to air, enhancing light extraction efficiency. This method can be used in other optical devices, such as organic LEDs, solar cells, and laser diodes, because of easy fabrication with large-area capability.

## Conflicts of interest

There are no conflicts to declare.

## Supplementary Material

RA-008-C8RA06773B-s001
