# Supplementary material for: Nano-imprinting of refractive-index-matched indium tin oxide sol–gel in light-emitting diodes for eliminating total internal reflection
Source: RSC Adv. 2018 Nov 1;8(65):37021–7. doi: 10.1039/c8ra06773b (PMC9089158; doi:10.1039/c8ra06773b)
Supplement: RA-008-C8RA06773B-s001 [file RA-008-C8RA06773B-s001.pdf]

Electronic Supplementary Material (ESI) for RSC Advances.
This journal is © The Royal Society of Chemistry 2018

Electronic Supplementary Information

Nano-imprinting of refractive-index matched indium tin oxide sol-gel in light-emitting diodes for eliminating total internal

Sungjoo Kim,[‡a] Chul Jong Yoo,[‡b] Jae Yong Park,[‡a] Sangwon Baek,[a] Won Seok Cho,[b] and Jong-Lam Lee[*a,b]*

[a.] Department of Materials Science and Engineering, Pohang University of Science and Technology (POSTECH), Pohang, 790-784 (Korea).

[b.] Division of Advanced Materials Science and Department of Materials Science and Engineering, Pohang University of Science and Technology (POSTECH), Pohang, 790-784 (Korea).

* E-mail: jllee@postech.ac.kr

‡ These authors contributed equally to this work.

Supplementary figures, Fig. S1-S4

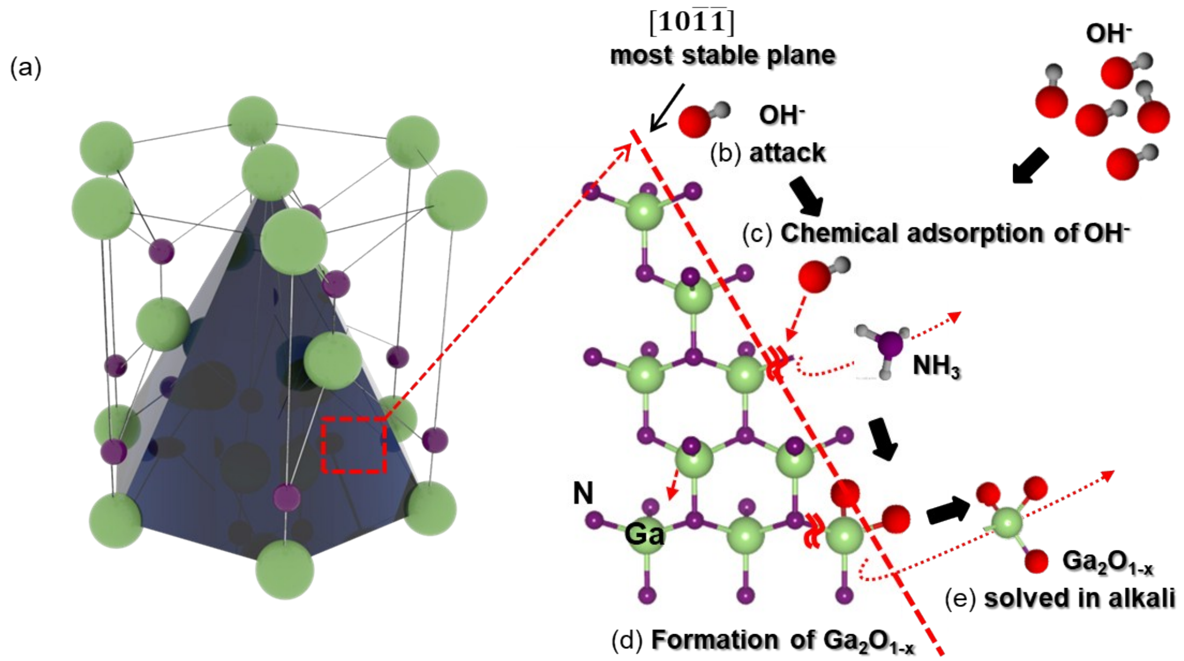

Figure S1. (a) Schematic illustration of photochemical etching of N polar GaN surface. (b) Nitrogen terminated layer with one negatively charged dangling bond on each nitrogen atom; (c) adsorption of hydroxide ions; (d) formation of oxides; (e) dissolving the oxides.

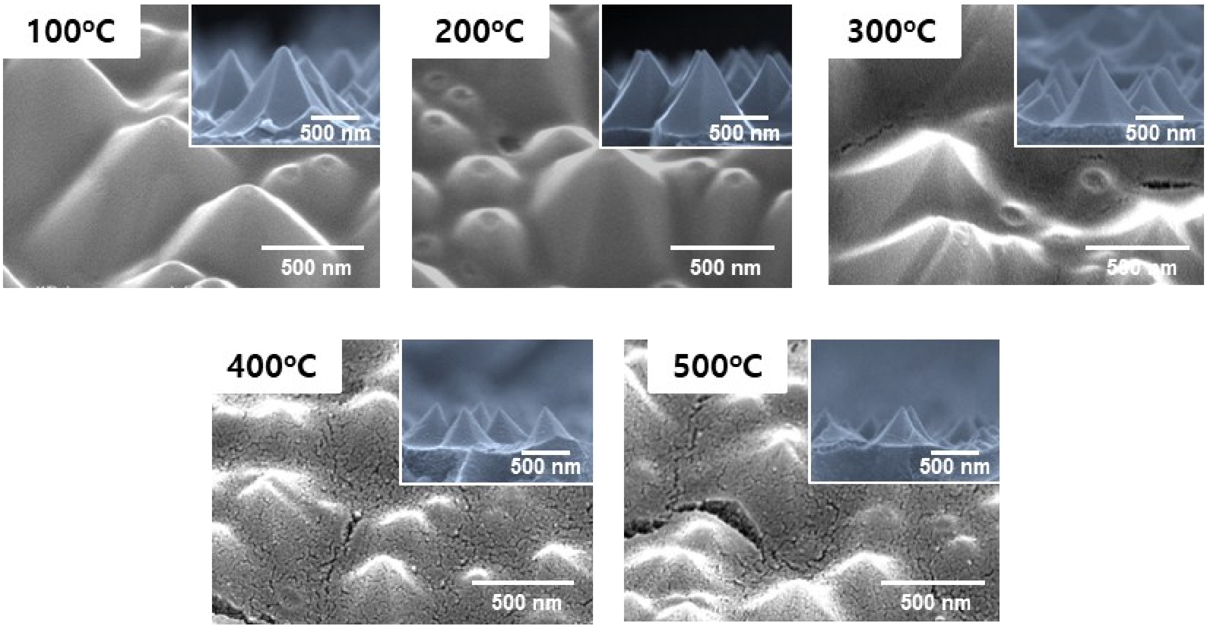

Figure S2. SEM images of nano-imprinted ITO sol-gel surfaces, as a function of the annealing temperature. The inset shows the cross-sectional images.

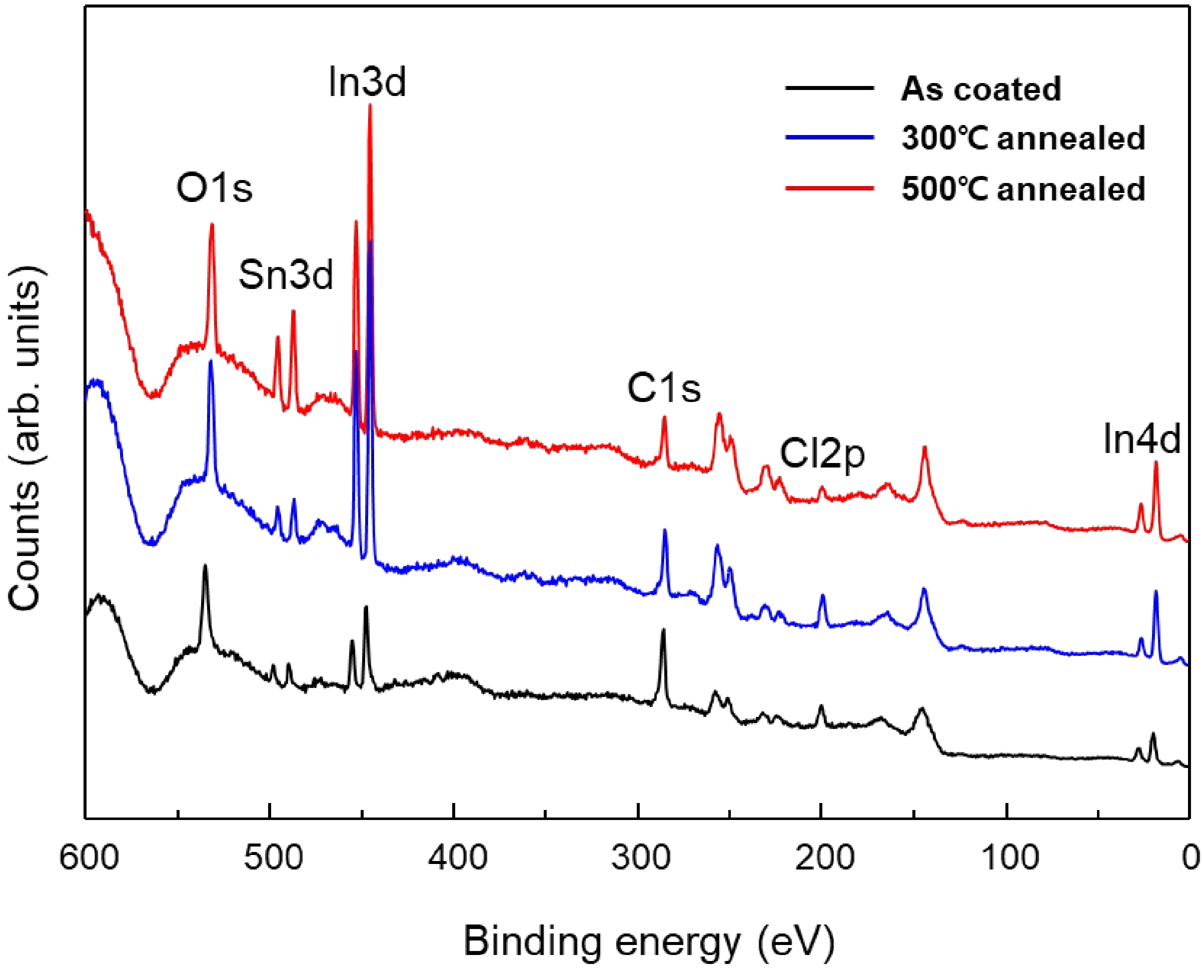

Figure S3. X-ray photoelectron spectra (wide scans) of as-coated, 300°C, and 500°C annealed ITO sol-gel films

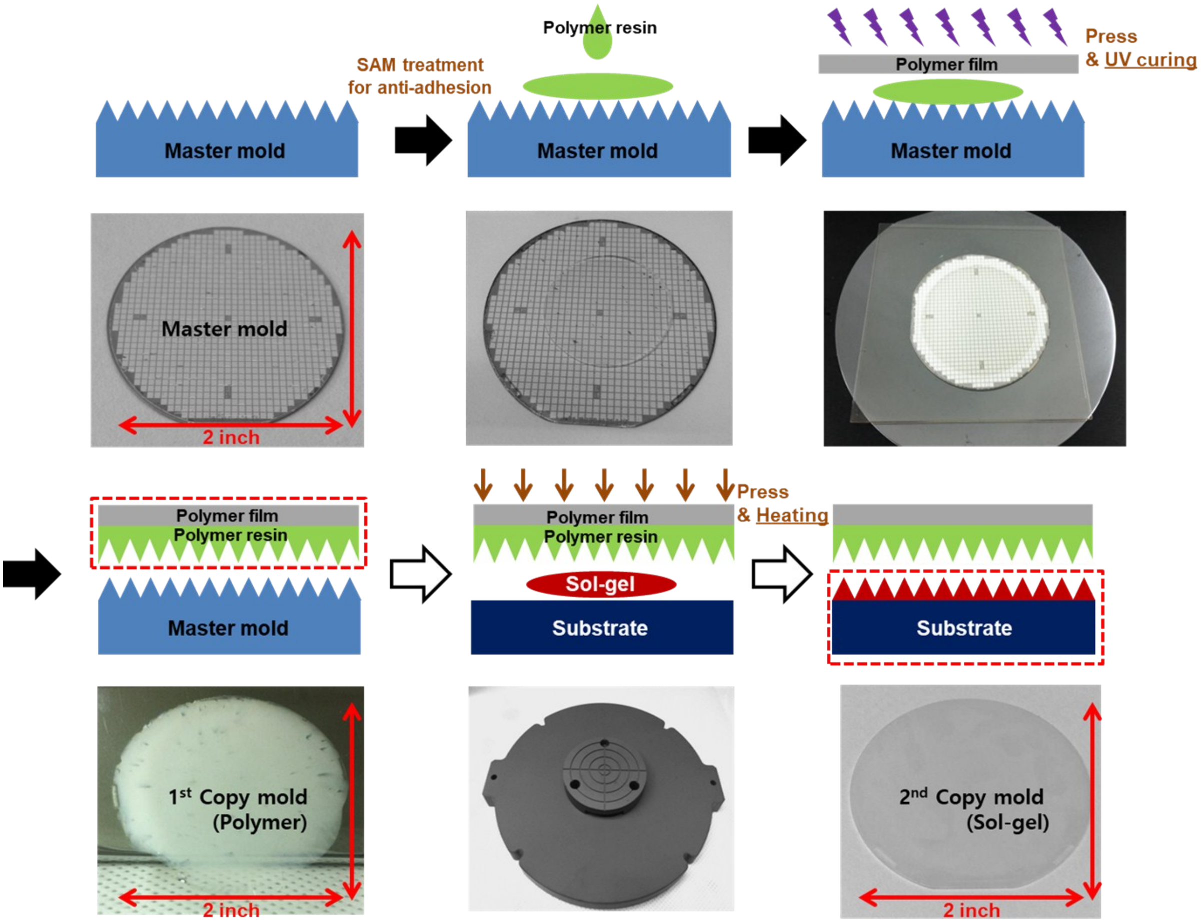

Figure S4. Fabrication flow of ITO sol-gel imprint, expressed as schematic illustrations and photographs.
